# The Potential Benefits of Chromium and Selenium Supplementation Across Psychiatric Disorders and Symptoms

**DOI:** 10.1097/JCP.0000000000002014

**Published:** 2025-05-12

**Authors:** Alessandro Di Lisi, Asia Dalla Valle, Marco Menchetti, Concetta Crisafulli, Chiara Fabbri

**Affiliations:** ^1^ Department of Biomedical and NeuroMotor Sciences, University of Bologna, Bologna, Italy; ^2^ Department of Biomedical and Dental Sciences and Morphofunctional Imaging, University of Messina, Messina, Italy

**Keywords:** trace elements, precision medicine, nutrition, psychiatric disorders, mental health

## Abstract

**Background:**

The conception of psychiatric disorders as systemic diseases implies an important role of nutrition in mental health. Essential trace elements are present in very small amounts in the body, yet they are essential for many physiological functions.

**Methods:**

Little is known about the potential role of chromium and selenium supplementation in mental disorders and other populations. To contribute to filling this gap, this review is focused on the possible benefits of chromium and selenium, alone or in combination with pharmacological treatments, in the treatment of mental disorders or psychiatric symptoms.

**Results:**

Chromium and selenium show promising effects on psychiatric symptoms across mental disorders. Selenium may have benefits on anxious-depressive symptoms in healthy populations and groups at risk of nutritional deficits and psychiatric symptoms, for example, women in the post-partum. In addition, chromium was linked to improved insulin sensitivity and glucose metabolism, which can benefit not only metabolic health but also mood regulation, with specific benefits on atypical symptoms of depression, such as appetite/weight gain. Selenium plays a role in reducing oxidative stress and inflammation, which may suggest positive effects on psychopathology and other conditions linked to these mechanisms.

**Conclusions:**

Both trace elements may affect positively mental health and cardio-metabolic-inflammatory conditions, which have a strong link with psychiatric disorders and their prognosis. Despite the evidence is not conclusive, chromium and selenium supplementation are interesting options to investigate in future research on the personalization of treatment in mental disorders.

The relationship between nutrition and mental health has gained increasing attention in recent years, including the role of trace elements. Trace elements are present in very small quantities in the body, but they play critical roles in numerous biological processes, and their importance in maintaining health cannot be overstated, as both deficiencies and excesses can lead to significant health problems.^1^

Essential trace elements include trace elements that are crucial for physiological functions and health.^2^ These consist of zinc (Zn), chromium (Cr), copper (Cu), selenium (Se), molybdenum (Mo), and iodine (Io).^2^ Both trace elements' excess and deficiency can be related to the development of psychiatric symptoms, explaining the use of some of them as augmentation to psychotropic medications. For example, zinc supplementation was shown to improve symptoms of depression^3,4^ while copper elevated serum concentrations can influence biochemical processes in critical brain regions and lead to neuronal dysfunction in major depressive disorder (MDD).^5^

Previous systematic reviews and meta-analyses examined the role of essential trace elements, focusing on the role of zinc deficiency and supplementation particularly in depression and schizophrenia. Zinc supplementation has been shown to potentially increase the efficacy of antidepressants, especially in treatment-resistant depression (TRD), by modulating neurotransmitter systems and potentially reducing inflammation.^6^ In schizophrenia, zinc augmentation to antipsychotics may increase benefits on both positive and negative symptoms.^7^

While the most studied essential trace element in relation to psychopathology is zinc, iodine was studied in relation to thyroid, metabolic, and developmental disorders^8^ and no previous research about molybdenum is available to the best of our knowledge. Copper deficiency can lead to a range of systemic abnormalities, but supplementation is not recommended unless there is a demonstrated deficiency, given the potential of toxicity, limiting the possibility of clinical trials or studies in the general population.^9^ Despite not being included among essential trace elements,^2^ rubidium has been the subject of some studies in the 70s–80s, for potential therapeutic effects in bipolar disorder.^10^ Rubidium was reported to show contrasting effects to lithium, for example, in terms of neurophysiological and behavioral effects in animals, and it was suggested as a possible antidepressant, with potential modulating effects on response to lithium in bipolar patients.^11^ However, results were obtained in small samples and were not conclusive; for this reason and for the fact that is not classified among essential trace elements,^2^ rubidium is no further discussed in this review.

Going back to essential trace elements, to the best of our knowledge, reviews summarizing and discussing the evidence regarding chromium and selenium supplementation across mental disorders are lacking. Chromium can be found in many foods, including meats, grain products, fruits, vegetables, nuts, spices, and brewer's yeast.^12^ In adults, the recommended intake is 30–35 micrograms/day for men and 20–25 micrograms/day for women,^12^ but dietary chromium absorption is low, ranging from about 0.4%–2.5%.^12^ Blood chromium healthy reference range was reported to be 0.7–28.0 μg/L^13^; however, this range is quite wide and there is no consensus on an internationally accepted range in the general population, other than variability depending on the geographical region,^14^ making challenging to understand what “low levels” are. Clinically evident chromium deficiency is extremely rare and has not been observed in healthy individuals on a typical diet.^15^ In humans, chromium plays a critical role in the metabolism of carbohydrates and fats, by enhancing the action of insulin, as first discovered in 1955 in a rat model of impaired glucose tolerance.^16^ This finding was confirmed in humans by different studies showing that chromium can reduce the risk of impaired glucose tolerance and improve glycemic control, especially in case of type 2 diabetes mellitus or glucose intolerance.^17,18^

Selenium is a semimetallic trace element essential for numerous physiological functions. The recommended dietary intake of selenium is 55 μg/day −70 μg/day in adults, and primary dietary sources include Brazil nuts, tuna, sardines, and shrimp.^19,20^ Selenium absorption in the intestinal tract is high; therefore, deficiency is rare on a typical diet, however some groups are more vulnerable (eg, people with alcohol use disorders, people suffering from malnutrition or malabsorption, pregnant women, patients with cardiovascular or oncological diseases, hepatic, and renal impairment).^21,22^ Measurements of plasma and urinary levels seem to be poor indicators of selenium deficiency, although there is a positive correlation between selenium intake and blood selenium levels, and the reference blood range was reported to be 100–300 μg/L^23–25^ However, other studies reported average blood levels outside of this interval, with protective effects toward depressive symptoms of values lower than this interval (∼82–85 μg/L),^26^ suggesting that there is no clear cutoff. In the body, selenium is present in inorganic forms as selenate and in organic forms as components of the amino acid selenocysteine,^27^ which is incorporated into seleno-proteins. Selenium's functions in the body are primarily related to its antioxidant properties, as seleno-proteins act as antioxidant enzymes and regulate transcription factors involved in the antioxidant response.^28^ Selenium plays a significant role in the brain's antioxidant system. The selenium content in the human brain is around 90–110 ng/mg wet weight, and when selenium is depleted, brain selenium levels are maintained even at the expense of other organs, and it is the first to be replenished upon reintroduction.^29^ Selenium exerts neuroprotective and neuroplastic roles, preventing neurodegeneration.^30^ Consistently, seleno-protein deficiency is associated with neurodegenerative diseases^31^ and may be implicated in intractable seizures.^32^ Selenium may be also linked to neurodevelopment, as selenium deficiency during pregnancy was associated with traits linked to attention-deficit hyperactivity and autism spectrum disorders in the offspring.^33^ Beyond its antioxidant role, selenium is also vital for thyroid function, immune regulation, and cardiovascular health.^34,35^

Given the physiological functions of chromium and selenium and the possibility that they may have beneficial effects on mental health, this review aimed to critically summarize previous studies on the topic, by considering the clinical effects of chromium and selenium supplementation in relation to psychopathology manifestations. We did not restrict our review to a specific mental disorder, but we considered the potential effects of these trace elements in any psychiatric condition, as well as on psychiatric symptoms in samples from the general population or specific groups at risk of nutritional deficits. This choice was motivated by the hypothesis that psychiatric symptoms represent dimensions that vary within a continuum and have trans-diagnostic value, as the same symptom dimension is likely influenced by similar biological dysfunctions across various diseases and in the general population, in line with the principles of precision psychiatry.^36^

## METHODS

We searched PubMed and APA PsychInfo from inception to June 2024 using combinations of the following search words: chromium, selenium, trace elements, supplementation, supplements, psychiatric disorders, depression, mood disorders, bipolar disorder, schizophrenia, psychotic disorders, anxiety disorders, eating disorders, posttraumatic, stress-related disorders, and neurodevelopmental disorders.

We included articles that satisfied the following criteria: 1) they investigated the clinical effects of chromium or selenium supplementation, either alone or in combination with other supplements or drugs, using a randomized controlled or open-label design; 2) they measured the clinical effects of supplementation by using standard psychopathological scales; 3) they included patients with any psychiatric disorder or evaluated psychopathological symptoms in samples without psychiatric disorders; 4) they were written in English. From each included article, we extracted the following: study design, year of publication, sample size, average age and sex distribution, diagnosis, diagnostic criteria, type of supplement used, additional treatments if any, dosage of the supplementation, psychopathological scales employed to measure outcomes, and a summary of results.

Despite not being systematic, this review aimed to provide an overview and critical interpretation of the available literature on the topic of interest, linking the evidence provided by the included studies to possible hypotheses explaining the biological and genetic mechanisms behind the reported clinical effects of chromium and selenium. As this review was not systematic, we did not include, compare or analyze effect size measures, even though they could potentially provide additional information. This choice was motivated also by heterogeneity factors across studies that would make comparisons difficult (eg, in study design and duration, and population characteristics, which included samples with different psychiatric diagnoses but also samples without psychiatric disorders).

Artificial intelligence (AI) tools were not used in this work.

## RESULTS

### Characteristics of the Included Studies

We identified 22 articles of interest, 9 for chromium and 13 for selenium (Tables 1, 2, respectively).

**TABLE 1:** Description of the Characteristics of Studies on Chromium

References	Design	N	Age	Female (%)	Follow-Up	Diagnosis	Supplementation	Comedications	Rating Instruments	Main Findings
Brownley et al, 2013^37^	Double blind cross- over case series	6	34–41	6 (100%)	2 menstrual cycles	Premenstrual dysphoric disorder	Sertraline + placebo vs sertraline + Cr polynicotinate 400 μg/d	SSRIs (all cases), lithium (1 case)	HAM-D, CGI	Only descriptive results: improvement in depressive symptoms, particularly decrease in interest, difficulty concentrating, feeling overwhelmed, and lethargy
Davidson et al, 2003^38^	RCT	15	46.4	11 (73%)	8 weeks	Atypical MDD (DSM-IV)	Cr picolinate 400–600 μg/d vs Placebo	None (wash-out period 7–30 days depending on the drug)	HAM-D, CGI, SCL-90, SOSS	↑Better Response and remission, no difference for specific symptoms; no patient discontinued treatment or reduced the dose because of side effects
Docherty et al, 2005^39^	RCT	113	46	76 (69%)	8 weeks	Atypical MDD or dysthymia (DSM-IV-TR)	Cr picolinate 600 μg/d vs Placebo	None (wash-out period 2–5 weeks depending on the drug)	HAM-D, CGI, SCL-90	↑Higher Improvement in appetite/eating increase, carbohydrate craving, and diurnal variation of feelings; ↑Better Response and improvement in the subgroup of patients with high carbohydrate craving
Brownley et al, 2013^40^	RCT	24	36	19 (83%)	6 months (with visit at month 3)	Binge eating disorder (DSM-IV)	Cr picolinate 600 μg/d vs Cr picolinate 1000 μg/d vs Placebo	SSRIs	EDE-Q QIDS-SR16	↑Higher Improvement in fasting glucose with a dose-response effect, nonsignificant reductions in binge frequency, weight and symptoms of depression
McLeod et al, 1999^41^	Open Label, case series	5	NA	3 (60%)	Up to 15 months	Dysthymic Disorder (DSM-IV), antidepressant-refractory	Cr polynicotinate or picolinate 400 μg/d + antidepressants	SSRIs (4 cases), nortriptyline (1 case)	BDI	Description of antidepressant effects potentiation and remission of symptoms
McLeod & Golden, 2000^42^	Open Label, case series	8	48.4	2 (25%)	Up to 17 months	Refractory mood disorders (Bipolar type II; Dysthymic disorder; MDD)	Cr polynicotinate or picolinate 400–600 μg/d	None (medications were suspended)	GAF	Description of improved symptoms and overall functioning
Amann et al, 2007^43^	Open Label	30	/	14 (47%)	2 years	Treatment-resistant rapid cycling Bipolar Disorder (DSM-IV)	Cr chloride 600–800 μg/d + naturalistic pharmacological treatments	Naturalistic treatments, including mood stabilizers, antipsychotics, antidepressants	HAM-D, MADRS, CGI-BP, YMRS	Improvement in CGI-BP scores and number of affective episodes (no comparator group)
Hockney et al, 2006^44^	RCT	112	21–67	44 (39%)	3 months	Schizophrenia or schizoaffective disorder (DSM-IV)	Usual antipsychotic + Cr picolinate 400 μg/d vs usual antipsychotic + placebo	Antipsychotics	PANSS, HAM-D	No benefit on weight, HbA1c, or psychopathology (patients were in a stable phase of disease at inclusion)
Jamilian et al, 2019^45^	RCT	53	27.5	53 (100%)	12 weeks	PCOS	1000 mg/d carnitine plus 200 μg/d chromium picolinate vs placebo	None	BDI, DASS, GHQ-28	↑Higher Improvement in depressive-anxious symptoms, general health, inflammatory-oxidative markers and hormonal profiles

Age was reported as mean or range in years. Comedications is referred to reported concomitant psychotropic medications. N = sample size. The main findings are referred to the group receiving chromium supplementation unless otherwise specified, reporting statistically significant associations between supplementation and outcomes, with no reference to the effect size or its possible clinical relevance. BDI, Beck Depression Inventory; CGI, Clinical Global Impression; CGI-BP, Clinical Global Impression–Bipolar Version; EDE-Q, Eating Disorder Examination Questionnaire; GAF, Global Assessment of Functioning; GHQ-28, General Health Questionnaire-28; HAM-D, Hamilton Depression Rating Scale; MADRS, Montgomery-Åsberg Depression Rating Scale; PANSS, Positive and Negative Syndrome Scale; QIDS-SR16, Quick Inventory of Depressive Symptomatology–Self Report; SCL-90, Symptom Checklist-90; SOSS, Self-rated Severity of Symptoms Scale; YMRS, Young Mania Rating Scale.

**TABLE 2:** Description of the Characteristics of Studies on Selenium

References	Design	n	Age	Female (%)	Follow-up	Diagnosis	Supplementation	Comedications	Rating Instruments	Main Findings
Sarris et al, 2022 ^46^	Open label	28	38.5	13 (46%)	20 weeks	Treatment-Resistant Obsessive-Compulsive Disorder (TR-OCD) (DSM-V)	Selenium 113 μg/d + N-acetyl cysteine + L-theanine + Zinc+ Magnesium + Pyridoxal-5′ phosphate + existing psychotropic medication	Naturalistic treatments, including antidepressants antipsychotics, benzodiazepines	Y-BOCS, DOCS, SIGHD-17, BAI, SDS, CGI, PGI, WHOQOL-BREF	Improvements in OC symptoms and all secondary outcomes (but no comparator group)
Sayyah et al, 2018^47^	RCT	32	35–38	9 (28%)	6 weeks	TR-OCD (DSM-V)	Selenium 200 μg/d + existing psychotropic medication + probiotics vs Placebo + existing psychotropic medication	SSRIs	Y-BOCS	↑Higher Improvement in Y-BOCS both for obsessions and compulsions
Jamilian & Ghaderi, 2021^48^	RCT	60	18–60	NA	12 weeks	Schizophrenia (DSM-IV-TR)	Selenium yeast 200 μg/d + probiotics vs Placebo	NA	HAM-D, CGI, SCL-90	↑Higher Improvement in PANSS and antioxidant capacity; higher reduction in CRP, fasting glucose and insulin
Ille et al, 2007^49^	RCT	40	46.4	32 (80%)	4 weeks	MDD (DSM-IV)	Selenium, ß-carotene, vit. C, E, B1, B2, B6, B12, folic acid, pantothenic acid, nicotinamide, zinc, magnesium, amino acid mixture + mirtazapine vs mirtazapine + placebo	Mirtazapine	HAM-D, BDI, GAF, CGI	↑Higher Improvement in symptoms and response, but not remission; no difference in the levels of amino acids, except taurine
Benton & Cook, 1991 ^50^	RCT (cross-over design)	49	14–74	33 (67%)	Two periods of 5 weeks	General population	Selenium 100 μg/d vs placebo	None	Profile of Moods States	↓Reduction in Mood-anxious symptoms (dietary Se intake was considered)
Gosney et al, 2008^51^	RCT	73	>60	41 (56%)	8 weeks	Older adults in nursing homes	Selenium 60 μg/d + Vitamin C vs placebo	None	MADRS, HAD	Significant symptoms of depression (29%) and anxiety (24%) at baseline, supplementation showed benefits in reducing them
Tolonen et al, 1985^52^	RCT	15	76	11 (73%)	12 months	Geriatric population	Sodium selenate 8 mg, organic selenium 50 μg and alpha-tocopherol acetate 400 mg/d vs placebo	None	SCAG	↑Higher Improvement in depression, anxiety, self-care, alertness, emotional lability, motivation, initiative, hostility, interest in the environment, fatigue, anorexia, and general impression
Jamilian et al, 2018^53^	RCT	60	48–40	60 (100%)	12 weeks	Polycystic ovary syndrome	Selenium 200 μg/d + Probiotics vs Placebo	None	BDI, GHQ-28, DASS-10	↑Higher Decrease in BDI, GHQ-28, DASS-10, ↑higher Improvement in hormonal and inflammatory profile
Mokhber et al, 2011^54^	RCT	85	16–35	85 (100%)	8 weeks	Pregnant women in the first trimester	Selenium 100 μg/d vs placebo until delivery	None	EPDS	↓Reduction in Depression scores
Shor-Posner et al, 2003^55^	RCT	63	39.5	31 (49%)	12 months	HIV/AIDS	Selenium 200 μg/d vs placebo	None	STAI, BDI, POMS	↑Higher Improvement in anxiety state and trait. No difference for depression or distress
Rayman et al, 2006^56^	RCT	501	60–74	Not Known	6 months	General population	Selenium 100–200–300 μg/d vs placebo	Antidepressants (n = 33)	POMS-BI, SF-36	No benefit on mood or quality of life
Hawkes & Hornbostel 1996^57^	RCT	11	20–45	0 (0%)	4 months	Men in a metabolic unit	Selenium 13–356 μg/d vs placebo	None	Profile of Mood States-Bipolar Form	No benefits on mood, but changes were correlated with initial Se concentration
Finley & Penland, 1998^58^	RCT	30	18–45	0 (0%)	105 days	General population	Selenium 32.6–226.5 μg/d vs placebo	None	POMS-BI, GVAS	↓Reduction in Depressive-anxious symptoms and ­ ↑increase in glutathione peroxidase activity

Age was reported as mean or range in years. N = sample size. The main findings are referred to the group receiving chromium supplementation unless otherwise specified. Comedications is referred to reported concomitant psychotropic medications. We reported statistically significant associations between supplementation and outcomes, with no reference to the effect size or possible clinical relevance. BAI, Beck Anxiety Inventory; BDI, Beck Depression Inventory; CGI, Clinical Global Impression; DASS-10: Depression Anxiety Stress Scales-10; DOCS, Dimensional Obsessive-Compulsive Scale; EPDS, Edinburgh Postnatal Depression Scale; GAF, Global Assessment of Functioning; GHQ-28, General Health Questionnaire-28; HAD, Hospital Anxiety and Depression Scale; HAM-D, Hamilton Depression Rating Scale; OC, obsessive-compulsive; NA, not available; SCL-90 = Symptom Checklist-90; MADRS, Montgomery-Åsberg Depression Rating Scale; PANSS, Positive and Negative Syndrome Scale; PGI, Patient Global Impression; POMS, Profile of Mood States; SCAG, Sandoz Clinical Assessment Geriatric Scale; SDS, Sheehan Disability Scale; SF-36, Short Form (36) Health Survey; SIGHD-17, Seventeen-item Hamilton Depression Rating Scale; STAI, State-Trait Anxiety Inventory; Y-BOCS, Yale-Brown Obsessive-Compulsive Scale; WHOQOL-BREF, World Health Organization Quality of Life scale.

For studies on chromium, the number of participants ranged from 5 to 113 individuals. Most studies included patients with mood disorders, had a duration from 8 weeks to 2 years and used chromium picolinate supplements, with dosages ranging from 200 μg to 1000 μg per day, as these are well tolerated with no evidence of specific side effects,^59^ while high doses may have adverse effects particularly in individuals with renal and/or liver impairment.^12^ Five studies combined chromium with psychopharmacological drugs and five studies were randomized controlled trials (RCTs).

For studies on selenium, the number of participants ranged from 11 to 501 individuals, with a range of different diagnoses (including obsessive-compulsive disorder, depression, schizophrenia) or healthy individuals; 12 studies were RCTs, and 4 studies combined selenium with psychopharmacological drugs. The doses of selenium supplementation ranged between 13 and 356 μg/d. The follow-up period was between 8 weeks and 1 year.

### Chromium: Clinical Effects on Affective Symptoms

Chromium supplementation has been most analyzed in relation to the potential effects on depressive symptoms across mental disorders, showing possible benefits, although there is a limited number of RCTs, and most studies have small sample sizes. The interventions, target populations, and outcome measures differed substantially among studies, making challenging to draw definitive conclusions (Table 1).

Two RCTs included patients with atypical unipolar depression, based on the evidence that chromium is involved in the metabolism of carbohydrates and enhances the action of insulin.^38,39^ The first study found higher rates of response and remission in the group receiving the supplementation versus placebo. While the second RCT did not found differences in the whole sample, it reported better response when considering the subgroup of patients with high carbohydrate craving, particularly for symptoms linked to the atypical spectrum (appetite increase/overeating, carbohydrate craving, diurnal variation of feelings).

A possible effect of chromium on symptoms of the atypical depressive spectrum and carbohydrate metabolism was suggested by other 2 studies, which considered mood symptoms and eating patterns/metabolic measures in individuals with premenstrual dysphoric disorder (PDD) and binge eating disorder (BED), respectively. These findings supported specific benefits of chromium supplementation on symptoms of reduced interest, difficulty concentrating, feeling overwhelmed, and lethargy in PDD,^37^ and improvement in fasting glucose in BED.^40^ The latter study reported nonsignificant reductions in binge episodes frequency and weight, suggesting possible metabolic benefits that are not necessarily the consequence of an improvement in eating patterns. However, in a sample with schizophrenia/schizoaffective disorder chromium supplementation to antipsychotic therapy had no benefits on weight or glycated hemoglobin.^44^ Compared to the study on BED, this latter study had a shorter duration (3 vs 6 months), which may explain the inconsistent findings, other than the prescription of antipsychotic drugs, given their known metabolic side effects.^60^ No benefits on psychotic and depressive symptoms in schizophrenia/schizoaffective disorder were found by the same study, although a substantial clinical stability at baseline and ongoing antipsychotic treatments could have played a role.

An improvement in inflammatory-oxidative markers and hormonal profiles alongside with mental health was reported by a RCT in women with polycystic ovary syndrome, when carnitine plus 200 μg/d chromium picolinate was taken for 12 weeks versus placebo.^45^ Specifically, the study found improvements in depressive-anxious symptoms in the experimental group but did not investigate the lipidic/glycemic profile.

Finally, 3 open-label studies with no control group evaluated chromium supplementation in patients with treatment-resistant mood disorders. Two case series described positive effects on mood and functioning in patients with dysthymic and refractory mood disorders when chromium was combined with antidepressants. One of the studies included 5 patients with antidepressant-refractory dysthymic disorder and described remission of affective symptoms when chromium was added to antidepressants as supplementation.^41^ The second case series included eight patients with refractory mood disorders who showed improvements in depressive symptoms and overall functioning with chromium supplements.^42^ The third study examined Cr supplementation in 30 patients with treatment-resistant rapid-cycling bipolar disorder, and the results suggested an improvement of symptoms and reduction in the number of affective episodes after 1 year, although only 7 patients remained in the study at the 1-year time point and results are therefore very preliminary.^43^

Chromium supplementation appears to be well tolerated, with studies reporting minimal side effects such as insomnia, headache, intense dreaming, and mild psychomotor activation.^42,43^

In conclusion, preliminary evidence suggests that chromium supplementation may improve mood symptoms, particularly those linked to the atypical spectrum, as these are associated with metabolic dysfunction, and it could be useful as add-on to pharmacological therapy in treatment-resistant mood disorders, with a good tolerability profile at least in the short term.

### Selenium and Potential Effects on the Anxious-Depressive and Obsessive Spectrum

Selenium supplementation showed promising preliminary effects on anxious-depressive and obsessive symptoms across various mental disorders, healthy samples, and patients with nonpsychiatric diseases (Table 2).

Two RCTs on samples with schizophrenia or MDD reported encouraging benefits, not just on psychopathology, but also on markers of oxidation-inflammation and glycemic profile.^48,49^ Both studies randomized patients to pharmacological treatment + selenium (combined with other supplements in^49^ see Table 2) or placebo, and they reported a greater improvement of symptoms in the selenium arm, which involved positive and negative symptoms in schizophrenia^48^, and overall depressive symptoms in patients with MDD.^49^ The RCT in patients with schizophrenia also found an increase in total antioxidant capacity and glutathione (GPH) activity, a reduction in C-reactive protein (CRP), and improvements in insulin homeostasis in the selenium arm. If these results are confirmed, add-on strategies with selenium could be beneficial also on immune-metabolic markers, with a potential protective effect on cardio-metabolic diseases, which are among the most common medical comorbidities in schizophrenia and depression.^61^

One open-label study^46^ and 1 RCT^47^ investigated selenium supplementation in patients with treatment-resistant obsessive-compulsive disorder (TR-OCD), suggesting positive clinical effects. The first study used a combination of nutraceuticals including selenium combined with antidepressants, while the RCT compared selenium + selective serotonin reuptake inhibitors (SSRI) versus placebo + SSRI. Both studies found a benefit of selenium on OCD symptoms, and the open-label study also reported a reduction in depressive-anxious symptoms, particularly in patients with a lower baseline severity of OCD symptoms, and an improvement in quality of life. Importantly, depressive and anxiety symptoms are frequent in OCD and make pharmacological and psychosocial treatment more difficult.^62^ However, it should be noted that only 1 RCT was available, and it combined selenium supplementation with probiotics in the experimental arm (Table 2); therefore, these results should be considered as preliminary, and independent confirmation is needed.

Several studies investigated selenium intake effects on anxious-depressive symptoms in healthy populations or vulnerable groups, providing potential implications for the reduction of these symptoms.

The first study to hypothesize possible psychological consequences of low selenium levels examined a sample from the British population.^50^ In this population, selenium intake was associated with a significant improvement in anxiety, depression, and mental vigilance, and basal selenium levels were inversely correlated with symptom severity. Similar results were reported in a small US sample, which suggested that a high versus low (226.5 vs 32.6 μg/d) selenium diet was associated with improvement in depressive-anxious symptoms, and platelet GPH peroxidase activity (GPx).^58^ Therefore, selenium supplementation may be beneficial on anxious-depressive symptoms in case of low-selenium diet/selenium deficiency. This hypothesis can explain at least partly the contrasting findings reported by 2 other studies, 1 performed in another sample from the UK general population^56^ and the other in a sample recruited in a metabolic research unit.^57^ Both studies showed no detectable clinical benefits on any of the mood scales in relation to selenium supplementation. However, the second study suggested a correlation between low selenium levels at baseline, the severity of mood symptoms and degree of their improvement, confirming the hypothesis that individuals with adequate selenium nutritional status do not benefit from selenium supplementation. The first study included healthy participants with relatively higher mean baseline selenium status compared to other data on the UK population (plasma concentration of 92 μg/L vs 79 μg/L),^56^ suggesting that seleno-proteins may already be adequately supplied with selenium at baseline, with a possible plateau effect on mood-anxiety symptoms. While this hypothesis is interesting, the available studies provided contrasting results as discussed; therefore, the possible moderating effect of baseline selenium nutritional status and other confounders should be clarified in future studies.

Other studies were performed in frail populations and other groups at risk of metabolic-nutritional unbalances as well as anxious-depressive symptoms. Two studies performed in older adults showed benefits on depressive scores, particularly anhedonia,^51^ and consistently, motivation, initiative, and interest in the environment.^52^ Cognitive and neurovegetative symptoms (mental alertness, fatigue, and anorexia) also improved, as well as emotional lability and anxiety, with a reflection on improved self-care.^52^ Benefits on depressive symptoms were found only in participants with baseline selenium levels <1.2 μM,^51^ and interestingly, they could continue for up to 1 year, parallel to an increase in average GPx activity.^52^

Improvements in inflammatory-oxidative parameters during selenium supplementation were examined in women with PCOS (polycystic ovary syndrome), alongside hormonal profiles and mental health.^53^ Co-administration of probiotics and selenium resulted in a significant improvement in depression-anxiety and stress scores compared to placebo, reduced CRP and malondialdehyde values, and increased GPH levels, in parallel to hormonal and metabolic improvements (eg, in dyslipidemia and insulin sensitivity).

Finally, benefits of selenium supplementation were shown in other 2 groups exposed to the risk of nutritional unbalances and anxious-depressive symptoms, namely, women during the post-partum and HIV+ individuals with drug use disorders. During the 8 weeks following delivery, depressive symptoms were lower in women who received selenium,^54^ suggesting it may be useful for the prevention of postpartum depression. On the other hand, in HIV+ drug users, nutritional chemoprevention with selenium increased vigor and improved anxiety compared to placebo, while no improvement in depression was observed.^55^

In conclusion, selenium supplementations may be beneficial on symptoms of the anxious-depressive and obsessive-compulsive spectrum, both in people with psychiatric disorders and in groups from the general population with low baseline selenium levels and at risk of nutritional deficits. In this regard, baseline determination of selenium levels can be useful, also considering that responses to excess selenium and factors involved in potential toxicity are only partially understood.^63^ In this regard, alopecia is an early and a well-established adverse effect of excess selenium exposure.^63^

## DISCUSSION

### The Clinical Effects of Chromium and Possible Biological Mechanisms

Even if the available evidence is not conclusive and has some limitations as previously mentioned, chromium supplementation may have potential benefits on psychiatric symptoms, possibly through mechanisms involving increased serotonergic activity, improved insulin sensitivity, and glucose metabolism. Given the limited number of studies, small sample sizes, and limitations in study design, further research is needed to confirm these findings and establish more definitive conclusions.

Chromium plays a crucial role in fat and glucose metabolism^17^ and it may modulate serotonergic neurotransmission by increasing the effects of insulin activity, which results in increased tryptophan availability.^64^ An increase in peripheral and central levels of tryptophan and brain serotonin was demonstrated after chromium supplementation in an animal model.^65^ This increase is also followed by a reduction in corticosterone response to the serotonin precursor 5-hydroxytryptophan (5-HTP), reducing the sensitivity of serotonin receptors, particularly 5-HT2A receptors. The reduction in 5-HT2A receptor sensitivity observed in animal and human studies following chromium supplementation suggests a down-regulation of these receptors, similar to the effects observed with some antidepressant drugs.^65^ Studies in humans did not show changes in peripheral tryptophan availability, but they demonstrated that chromium lowers the cortisol response to 5-HTP, indicating a potential central modulation of serotonin neurotransmission, and a reduction in the hyperactivity of stress response associated with depression.^64^

The effects on metabolism, stress response, and serotonin pathways explains the possible benefits of chromium on depressive symptoms linked to the atypical spectrum, such as increased appetite/weight, carbohydrate craving and fatigue, with a potential benefit on glucose metabolism. There is a strong bidirectional link between mood and metabolic disorders, such as type 2 diabetes mellitus, reflecting their high comorbidity and probable reciprocal causal effects.^66^ These conditions also negatively impact each other, for example. in terms of response to treatments and prognosis^66^; therefore therapeutic approaches that considered both mental and metabolic health have fundamental importance.

Another interesting observation is the association between atypical neurovegetative symptoms, increased systemic inflammation and risk of TRD.^67^ Chronic low-grade inflammation and metabolic dysfunctions are common in patients with atypical neurovegetative symptoms, which include hyperphagia, weight gain, hypersomnia, and fatigue.^68^ Therefore, the identification or repurposing of therapeutics that are effective in this group of patients may prevent disease chronicity and development of cardio-metabolic diseases. For example, patients with higher body mass index and CRP may respond worse to SSRIs, which are typically the first line treatment for depression,^69 70^ underlying the importance of treatment personalization in this group.

As discussed, atypical symptoms are linked to the risk of poor response to standard antidepressants and treatment resistance; despite there are no previous RCTs, 2 case series suggested potential benefits of chromium in treating refractory mood disorders, in combination with antidepressants, or even after their interruption.^41,42^ A third open-label study suggested benefits in patients with treatment-resistant rapid cycling bipolar disorder, but it was not able to perform convincing analyses due to the very high drop-out rate at year 1 of follow-up.^43^ The design and small sample size of these studies do not allow to trace conclusions, and future RCTs are needed to also clarify the tolerability profile and the optimal dose range of supplementation. Most studies used Cr polynicotinate 400–600 μg, and only 1 compared 600 μg versus 1000 μg, suggesting a dose-response effect on fasting glucose.^40^

Consistently with the results of studies performed in samples with mood disorders, chromium supplementation may be useful in eating disorders^40^ and other psychiatric disorders when symptoms of the atypical neurovegetative spectrum and/or metabolic dysfunction are present, leading to improvements not only in depressive symptoms but also in specific aspects related to appetite, eating behaviors, and metabolism. Preliminary evidence of improvement in inflammatory-oxidative markers in women with PCOS was also reported.^45^

### The Clinical Effects of Selenium and Possible Biological Mechanisms

Based on the available studies, no strong indication can be given about the use of selenium supplementation for treating psychiatric disorders or symptoms; the available evidence suggests that selenium supplementation may improve affective-anxious and obsessive-compulsive symptoms in people with psychiatric disorders, and other groups who may have reduced selenium levels due to nutritional factors or concomitant diseases or conditions. Even if the evidence is not conclusive due to limitations in study design and partially contrasting results across studies, the hypothesis of beneficial effects of selenium is supported by its positive impact on inflammatory-metabolic parameters, with potential advantages on medical comorbidities and on the side effects profile of some medications.

The primary biological mechanisms of selenium's beneficial effects are hypothesized to be the reduction of oxidative stress and the modulation of serotonergic and dopaminergic activity; in addition, selenium was also linked to thyroid health.^71–73^ Selenium is involved in the antioxidant system and plays an important role in increasing GPx activity, reducing interleukin-6 and TNF-α, and in the regulation of MAP kinase pathways/arachidonic acid metabolism, which leads to anti-inflammatory effects.^74^ Seleno-proteins catalyze enzymatic redox reactions and protect DNA from oxidative damage produced by inflammation.^75^ Selenium also has immunostimulatory functions, as it increases macrophage activity, immunoglobulin production and NK cell cytolysis.^57^ In addition, selenium is associated with increased synthesis of brain-derived neurotrophic factor, which is crucial for neuroplasticity, and with modulation of dopaminergic and serotonergic neurotransmission, by contributing to increased dopamine in the substantia nigra, and increased dopamine and serotonin turnover in the prefrontal cortex.^30^ Consistently, numerous studies demonstrated that seleno-proteins promote neurogenesis in the thalamus, brainstem, and hippocampus^76^ and that selenium supplementation restores neurogenesis.^77^

These biological mechanisms may explain the clinical benefits observed on psychopathology (Table 2), given the link of inflammation, oxidative stress, neuroplasticity and neurotoxic mechanisms with psychiatric conditions.^78–80^ Consistently, OCD was associated with dysfunction of the redox chain and increase in free radicals^81,82^ alterations which may be particularly prominent in TR-OCD, leading to glutamate-induced neurotoxicity, loss of hippocampal neurons and damage to glial cells.^83^

Selenium deficiency may be particularly relevant in pregnant women, as a mother's selenium requirement during pregnancy and lactation increases, due to selenium being selectively transported to the fetus, even at the cost of creating a deficiency in the mother.^84^ Therefore, selenium may have an important role in the prevention of postpartum depressive symptoms.^54,85,86^ It is also important to discuss the benefits of selenium on cardiovascular risk, a major cause of morbidity and mortality in patients with mental disorders, and more in general, conditions associated with inflammation, oxidative stress, and metabolic disturbances.^87,88^ Selenium indeed reduces fasting glycemia, insulin levels, and increases insulin sensitivity.^48^ It has potential hepatoprotective and nephroprotective effects, which may reduce some adverse effects of exogeneous substances.^28^

Finally, selenium is essential for the maintenance of thyroid health and thyroid hormone metabolism.^71–73^ The thyroid gland plays a critical role in mental health,^89^ and thyroid hormones are among the therapeutic augmentation options in treatment-resistant depression.^90^ Consistently, the effects of selenium deficiency on mood could be partly mediated by selenium deficiency-induced changes in thyroid function.^71^ The modulation of thyroid function may be particularly relevant in patients with PCOS,^91^ explaining the benefits found in this group of patients.^53^

As discussed for chromium, future RCTs should take in account also the tolerability profile and determine the optimal dose range of supplementation, considering baseline plasma levels of selenium. As last point, it is useful to note that all studies that investigated selenium supplementation in clinical samples used preparations including other supplements (Table 2); therefore, the exclusion of effects due to other supplements is not possible. On the contrary, studies performed in the general population or samples without psychiatric disorders compared selenium supplementation with placebo, supporting the specificity of the observed effects.

### Genetic Modulation of Selenium/Chromium Levels: Possible Links With Risk of Deficiency, Clinical Symptoms, and Response to Supplementation

As discussed, supplementation of essential trace elements may show benefits in individuals with reduced baseline levels, particularly in the case of selenium, even if there is no clear consensus on “normal” levels as discussed, and blood levels are probably a poor measure of other tissues reserves. The identification of individuals genetically predisposed to lower levels of essential trace elements may be useful to put in place preventive measures, as these people may need higher diet intake to maintain physiological concentrations. Genome-wide association studies have investigated the association between genetic variants and blood levels of selenium and chromium, and they may point to possible mechanisms behind the predisposition toward their deficit, the development of psychopathological symptoms and benefits deriving from supplementation.

Regarding selenium, significant genome-wide associations were found for polymorphisms in *SLC18A2*, *DMGDH*, *CPS1*, *BHMT2*, and *BHMT* genes.^92,93^ *SLC18A2* (Solute Carrier Family 18 Member A2) encodes for a transporter of amine neurotransmitters into synaptic vesicles (VMAT2), which genetic variation was associated with fear behavior in mice.^94^ Interestingly, mice with elevated VMAT2 show increased dopamine release, and improved outcomes on anxiety/depressive behaviors with neuroprotective effects.^95^ On the other hand, heterozygous mice for this gene show depressive-like behaviors, in line with the observation that reserpine (a VMAT2 inhibitor) is associated with depressive symptoms in humans^96^ and evidence of depressive symptoms and sleep disturbances within families carrying variants in this gene.^97^ These findings suggest a link between variation in the *SLC18A2* gene, predisposition to low selenium levels and depressive/anxious symptoms, and benefits of selenium supplementation on these symptoms, consistently with the observation that selenium exposure is associated with VMAT2 levels.^98^

*DMGDH* (dimethylglycine dehydrogenase), *BHMT2* (betaine-homocysteine S-methyltransferase 2), and *BHMT* (betaine-homocysteine S-methyltransferase) code for enzymes involved in sulfur-containing amino acid metabolism, which have not been linked to psychiatric symptoms/treatment response to the best of our knowledge, as well as *CPS1* (carbamoyl-phosphate synthase), a mitochondrial enzyme involved in the urea cycle.

For chromium levels, only 1 suggestive association has been reported, with rs12915189 in the *CRTC3* (CREB Regulated Transcription Coactivator 3) gene.^99^ *CRTC3* has a role in catecholamine signaling regulating glucose/fatty acid oxidation and energy balance,^100^ suggesting a potential link with atypical symptoms of depression involving appetite/eating behaviors. Interestingly, this gene has been reported to have a suggestive association with TRD^101^ and social behaviors in mice,^102^ supporting the importance of further research to clarify the effects of variants in this gene on chromium levels, specific psychiatric symptoms and response to treatments.

## CONCLUSIONS

Chromium and selenium show promising clinical effects on affective, anxious, and obsessive-compulsive symptoms in various mental disorders. Selenium had interesting effects also in samples from the general population and those at risk of nutritional deficits and psychiatric symptoms, suggesting that the evaluation of selenium dietary intake and plasma levels can be useful in these cases. However, as discussed, there is still no clear consensus about normal plasma levels of these essential trace elements, and future studies can help in providing definitive guidance.

Both trace elements can exert benefits on immune-inflammatory and metabolic parameters, potentially contributing to preventive effects with respect to the development of common medical comorbidities in psychiatric disorders. Chromium improves insulin sensitivity and glucose metabolism, which can benefit not only metabolic health but also mood regulation. Selenium acts as a cofactor in molecular mechanisms reducing oxidative stress, and it regulates inflammation and has a role in neuroprotective processes.

Because of the low number of studies available, the small sample size and the heterogeneity of methods and results across studies, it is however not possible to provide solid recommendations about the use of chromium and selenium supplementation in people with psychiatric disorders or symptoms. Given the heterogeneity of the available studies, this review did not provide a quantitative synthesis of results, which would be however important to produce cumulative and more solid evidence. Future studies should attempt to fill the gaps remaining in the literature, as essential trace elements supplementation is a potential useful option as enhancement to other therapies, which should be considered and adapted based on the individual characteristics, such as symptom profile, risk of nutritional deficits, and genetic predisposition.
